# Investigation of reproducibility of differentially expressed genes in DNA microarrays through statistical simulation

**DOI:** 10.1186/1753-6561-3-s2-s4

**Published:** 2009-03-10

**Authors:** Xiaohui Fan, Leming Shi, Hong Fang, Stephen Harris, Roger Perkins, Weida Tong

**Affiliations:** 1 National Center for Toxicological Research (NCTR), US Food and Drug Administration, 3900 NCTR Rd., Jefferson, AR 72079, USA; 2 Pharmaceutical Informatics Institute, College of Pharmaceutical Sciences, Zhejiang University, Hangzhou 310027, PR China; 3 Z-tech Corporation, an ICF International, National Center for Toxicological Research (NCTR), US Food and Drug Administration, 3900 NCTR Rd., Jefferson, AR 72079, USA

## Abstract

Recent publications have raised concerns about the reliability of microarray technology because of the lack of reproducibility of differentially expressed genes (DEGs) from highly similar studies across laboratories and platforms. The rat toxicogenomics study of the MicroArray Quality Control (MAQC) project empirically revealed that the DEGs selected using a fold change (FC)-based criterion were more reproducible than those derived solely by statistical significance such as P-value from a simple t-tests. In this study, we generate a set of simulated microarray datasets to compare gene selection/ranking rules, including P-value, FC and their combinations, using the percentage of overlapping genes between DEGs from two similar simulated datasets as the measure of reproducibility. The results are supportive of the MAQC's conclusion on that DEG lists are more reproducible across laboratories and platforms when FC-based ranking coupled with a nonstringent P-value cutoff is used for gene selection compared with selection based on P-value based ranking method. We conclude that the MAQC recommendation should be considered when reproducibility is an important study objective.

## Background

The utility of DNA microarrays has been demonstrated in clinical applications and risk/safety assessments [1-6]. With the wide variety of array platforms and analysis approaches, however, challenges remain in this field. For example, several publications [7-11] recently raised concerns about the reliability of microarray technology based on the lack of agreement in differentially expressed genes (DEGs) obtained from different laboratories and array platforms for highly similar study designs and experiments. By reanalyzing seven of the largest public DNA microarrays datasets aimed at cancer prognosis, Michiels et al. found that the signature genes of the classifiers were extremely unstable [11]. The MicroArray Quality Control (MAQC) project conducted a large study using reference RNA samples and a toxicogenomics dataset [12,13] revealed that the DEGs selected using fold change (FC)-based criterion were more stable in terms of reproducibility across labs and platforms than those derived solely from statistical significance measures such as P-value from simple t-tests. The MAQC study caused some to argue that the MAQC conclusion could be so broadly generalized. In response, this study sought to duplicate the finding of MAQC, except through statistical simulation using postulated datasets. Specifically, we generated a set of simulated microarray datasets with varying amount of noise, expression magnitude, and sample size in order to systematically compare the relationships among gene selection/ranking rules (i.e., P-value, FC and their combinations) with respect to reproducibility of DEGs.

## Methods

Two simulated groups of samples were generated, a control group and a treatment group. The control and treatment groups consisted of either 5 or 50 replicates (samples) with each replicate containing 12,000 genes. The gene intensities of the samples in the control group were simulated by Signal + Noise while the corresponding gene intensities of the treated samples were Signal + FC + Noise. Both Signal and Noise were distributed normally, while FC was distributed exponentially. The study used the set of parameters that are summarized in Table 1. Specifically, both treated and control groups contain either 50 or 5 simulated replicates with a distributed CV (coefficient of variation) similar to those observed in the MAQC study for the reference RNA samples and rat toxicogenomics dataset. CV values of 2%, 10%, 30%, and 100% were used, corresponding to low, medium, high, and very high noise level, respectively. For each CV value, three expression magnitudes were considered corresponding to mean FC of 1.5, 0.6 and 0.2; these values are corresponding to the MAQC's study for the reference RNA samples and rat toxicogenomics dataset as well as consistent with the range typically found in clinical microarray experiments, respectively.

**Table 1:** Summary of the parameters used in this study.

**CV**	Low	Medium	High	Very High
	
	~2%	~10%	~30%	~100%
**Magnitude** **(FC)**	MAQC main study	MAQC Rat toxicogenomics	Clinical application	
	
	~1.5	~0.6	~0.2	

**Sample Size**	5 per group		50 per group	

## Results and discussions

The study applied 24 simulated conditions (or 24 permutations) corresponding to two sample sizes, each having four values of CV and three different mean FC values, corresponding to Table 1. For each permutation, six gene selection methods were used to determine DEGs by comparing the treated group with the control group. These gene selection methods were (1) *FC*: genes are rank ordered by FC and DEGs determined by a FC cut-off only; (2–3) *FC (P < 0.01) *and *FC (P < 0.05)*: genes are rank ordered first by FC and DEGs are determined by a P-value cutoff of either 0.01 and 0.05; (4) *P*: genes are rank ordered by P-value from the simple t-test and DEGs are selected using a specified P-value cutoff; and (5–6) *P (FC > 1.4)*, and *P (FC > 2)*: genes are rank ordered first by P-value and DEGs are then determined by either a FC = 1.4 or FC = 2 cutoff. Each permutation was repeated twice to mimic the process of conducting the same experiment in two different labs or two different platforms. The resulting DEGs from two simulations were compared to assess reproducibility across labs or platforms based on the percentage of overlapping genes (POG).

**Figure 1:**
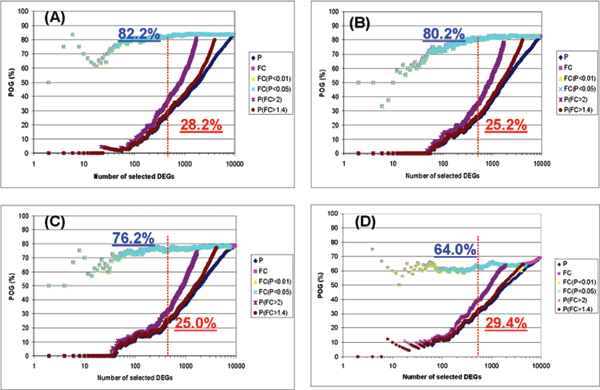
**The relationship of POGs with the degree of noise level in the simulated datasets**: (A) Low noise (CV = 2%); (B) Medium noise (CV = 10%); (C) High noise (CV = 30%); and (D) Very high noise (CV = 100%). The simulated datasets were set to the expression magnitude difference between the treated and control groups of 1.5 and the sample size of 50. The x-axis represents the number of genes selected as differentially expressed, and the y-axis represents the POG (%) of two gene lists for a given number of differentially expressed genes. Each line on the graph represents the overlap of differentially expressed gene lists based on one of six different gene ranking/selection methods. The red and blue numbers give the POG (%) for 500 selected DEGs (red dashed line) from P rank ordering only and FC rank ordering with P < 0.05, respectively.

Figure 1 compares six gene selection methods applied to four datasets, each containing a different noise level (i.e., CV = 2%, 10%, 30% and 100%), where POG is shown as a function of the number of genes selected as differentially expressed between two simulations for the same permutation (magnitude = 1.5 and sample size = 50). In general, the FC-based gene selection methods outperformed the P-based gene selection method in terms of DEG reproducibility measured by POG. Specifically, three FC-based gene selection methods, i.e. FC, FC (P < 0.01), and FC (P < 0.05) consistently result in the highest POG values, regardless of CV value. Higher noise consistently results in lower POG (i.e., DEG reproducibility), as expected. The POG consistently decreases with increasing CV. For P value selection methods, higher FC cutoff results in higher POG. All results are consistent with MAQC observations.

**Figure 2:**
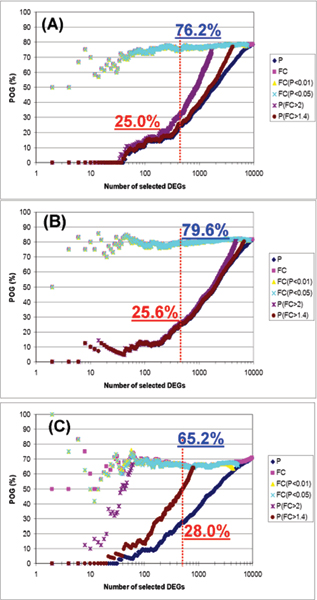
**The relationship of POG with the degree of difference in expression magnitude between the treated versus control groups.** (A) Magnitude = 0.6; (B) Magnitude = 1.5; and (C) Magnitude = 0.2. The simulated datasets had CV = 30% and sample size = 50. The x-axis represents the number of genes selected as differentially expressed, and the y-axis represents the POG (%) of two gene lists for a given number of differentially expressed genes. Each line on the graph represents the overlap of differentially expressed gene lists based on one of six different gene ranking/selection methods. The red and blue numbers give the POG (%) when 500 genes (red dashed line) are selected as DEGs using P rank ordering only and FC rank ordering with P < 0.05, respectively.

Figure 2 compares six gene selection methods on three datasets, each having a different magnitude level between the treated and control groups (i.e., FC = 1.5, 0.6 and 0.2). Similar to Figure 1, the FC-based methods resulted in greater reproducibility compared to the P-based method. Furthermore, POG increases with increasing differential expression magnitude for FC selection methods. However, this trend is not prominent for P value-based selection methods, where it seems that the trend is equivocal.

**Figure 3:**
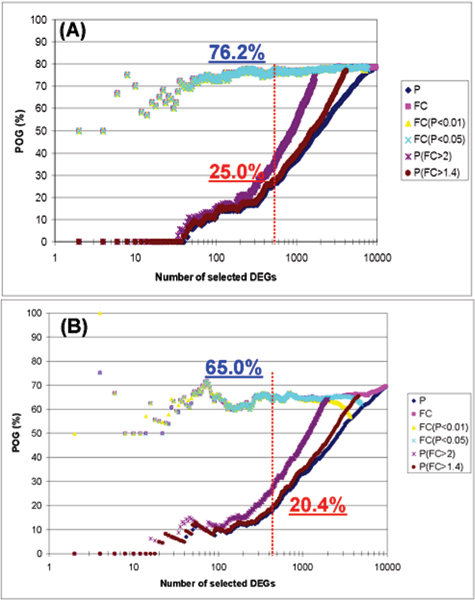
**The relationship of POG with the sample size: (A) 50 samples/group and (B) 5 samples/group**. The simulated datasets had CV = 30% and magnitude = 50 (see Table 1). The x-axis represents the number of genes selected as differentially expressed, and the y-axis represents the POG (%) of two gene lists for a given number of differentially expressed genes. Each line on the graph represents the overlap of differentially expressed gene lists based on one of six different gene ranking/selection methods. The red and blue numbers give the POG (%) when 500 genes (red dashed line) are selected as DEGs using P rank ordering only and FC rank ordering with P < 0.05, respectively.

Figure 3 compares six gene selection methods on two datasets, one having sample size of 50 and the other having sample size 5. FC-based methods again give higher POG than P value-based methods, with the larger sample size resulting in higher POG for either selection approach.

Whereas POG are affected by the degree of noise level, expression magnitude and sample size of the datasets, the above results clearly demonstrated that the DEGs become more reproducible, especially when fewer genes are selected, if the FC is included as the ranking criterion for subsequent DEGs identification. It is likely that the discordance of reported microarray results in literature is in large part due to the widespread of using P-based approach to rank genes over the FC-based method. The results of our another related study demonstrated that the relationship of the tradeoff between reproducibility and specificity/sensitivity in the FC (P) approach can be balanced by weighting the FC as a primary consideration in gene ranking: that is an FC criterion explicitly incorporates the measured quantity to ensure reproducibility, whereas a P criterion incorporates control of sensitivity and specificity [14].

## Conclusion

Our simulation results show that the choice of gene selection method significantly affects apparent reproducibility of DEGs as measured by POG. Reproducibility as measured by POG between lists substantially increases when FC is the ranking criterion for identifying DEGs, especially for shorter gene lists. This observation holds for different noise levels, expression magnitudes and sample sizes. Our simulation are consistent with MAQC's conclusion that to generate more reproducible DEG lists across labs and platforms, the FC ranking with a nonstringent P-value cutoff, so named the FC (P) approach, should be considered when reproducibility is a consideration in a microarray study.

## Competing interests

The authors declare that they have no competing interests.

## Authors' contributions

XF performed data analyses and finished the first draft of the manuscript. WT and HF guided the analysis and helped writing manuscript. LS had the original idea for statistical simulation. RP also helped manuscript writing. SH helped the microarray data management. All authors participated in preparation of the manuscript, and approved its final version.

## References

[B1] Rosenwald A, Wright G, Chan WC, Connors JM, Campo E, Fisher RI, Gascoyne RD, Muller-Hermelink HK, Smeland EB, Staudt LM (2002). The use of molecular profiling to predict survival after chemotherapy for diffuse large-B-cell lymphoma. N Engl J Med.

[B2] van't Veer LJ, Dai HY, Vijver MJ van de, He YDD, Hart AAM, Mao M, Peterse HL, Kooy K van der, Marton MJ, Witteveen AT (2002). Gene expression profiling predicts clinical outcome of breast cancer. Nature.

[B3] Bhattacharjee A, Richards WG, Staunton J, Li C, Monti S, Vasa P, Ladd C, Beheshti J, Bueno R, Gillette M (2001). Classification of human lung carcinomas by mRNA expression profiling reveals distinct adenocarcinoma subclasses. Proc Natl Acad Sci USA.

[B4] Pomeroy SL, Tamayo P, Gaasenbeek M, Sturla LM, Angelo M, McLaughlin ME, Kim JYH, Goumnerova LC, Black PM, Lau C (2002). Prediction of central nervous system embryonal tumour outcome based on gene expression. Nature.

[B5] Iizuka N, Oka M, Yamada-Okabe H, Nishida M, Maeda Y, Mori N, Takao T, Tamesa T, Tangoku A, Tabuchi H (2003). Oligonucleotide microarray for prediction of early intrahepatic recurrence of hepatocellular carcinoma after curative resection. Lancet.

[B6] Fan C, Oh DS, Wessels L, Weigelt B, Nuyten DSA, Nobel AB, van't Veer LJ, Perou CM (2006). Concordance among gene-expression-based predictors for breast cancer. N Engl J Med.

[B7] Marshall E (2004). Getting the noise out of gene arrays. Science.

[B8] Ioannidis JPA (2005). Why most published research findings are false. PLos Med.

[B9] Simon R (2006). Development and evaluation of therapeutically relevant predictive classifiers using gene expression profiling. J Natl Cancer Inst.

[B10] Ein-Dor L, Zuk O, Domany E (2006). Thousands of samples are needed to generate a robust gene list for predicting outcome in cancer. Proc Natl Acad Sci USA.

[B11] Michiels S, Koscielny S, Hill C (2005). Prediction of cancer outcome with microarrays: a multiple random validation strategy. Lancet.

[B12] Guo L, Lobenhofer EK, Wang C, Shippy R, Harris SC, Zhang L, Mei N, Chen T, Herman D, Goodsaid FM (2006). Rat toxicogenomic study reveals analytical consistency across microarray platforms. Nat Biotechnol.

[B13] Shi LM, Reid LH, Jones WD, Shippy R, Warrington JA, Baker SC, Collins PJ, de Longueville F, Kawasaki ES, Lee KY (2006). The MicroArray Quality Control (MAQC) project shows inter- and intraplatform reproducibility of gene expression measurements. Nat Biotechnol.

[B14] Shi L, Jones WD, Jensen RV, Harris SC, Perkins RG, Goodsaid FM, Guo L, Croner LJ, Boysen C, Fang H (2008). The balance of reproducibility, sensitivity, and specificity of lists of differentially expressed genes in microarray studies. BMC Bioinformatics.

